# The Most Important Metabolic Diseases in Dairy Cattle during the Transition Period

**DOI:** 10.3390/ani14050816

**Published:** 2024-03-06

**Authors:** Vincenzo Tufarelli, Nikola Puvača, Dragan Glamočić, Gianluca Pugliese, Maria Antonietta Colonna

**Affiliations:** 1 Department of Precision and Regenerative Medicine and Jonian Area (DiMePRe-J), Section of Veterinary Science and Animal Production, University of Bari Aldo Moro, 70010 Valenzano, Italy; gianluca.pugliese1997.mv@gmail.com; 2 Laboratory for Food Quality and Toxicology, Department of Engineering Management in Biotechnology, Faculty of Economics and Engineering Management, University of Business Academy in Novi Sad, 21107 Novi Sad, Serbia; 3 Department of Animal Science, Faculty of Agriculture, University of Novi Sad, Trg Dositeja Obradovića 8, 21000 Novi Sad, Serbia; dragan.glamocic@stocarstvo.edu.rs; 4 Department of Soil, Plant and Food Sciences, University of Bari Aldo Moro, 70125 Bari, Italy; mariaantonietta.colonna@uniba.it

**Keywords:** dairy cattle, diseases, milk, cows, dairy industry, metabolic disorders

## Abstract

**Simple Summary:**

This review delves into key metabolic diseases affecting dairy cattle such as subacute ruminal acidosis (SARA), ketosis, and hypocalcemia. The aim of this review was to examine each disease in terms of its etiology, pathophysiology, clinical manifestations, diagnostic approaches, and treatment and prevention strategies. This review emphasizes early diagnosis and proactive management, so it can serve as a valuable resource for veterinarians, researchers, and dairy farmers, offering insights into the prevention and treatment of these prevalent metabolic diseases in dairy cattle.

**Abstract:**

This review paper provides an in-depth analysis of three critical metabolic diseases affecting dairy cattle such as subacute ruminal acidosis (SARA), ketosis, and hypocalcemia. SARA represents a disorder of ruminal fermentation that is characterized by extended periods of depressed ruminal pH below 5.5–5.6. In the long term, dairy herds experiencing SARA usually exhibit secondary signs of the disease, such as episodes of laminitis, weight loss and poor body condition despite adequate energy intake, and unexplained abscesses usually 3–6 months after an episode of SARA. Depressed milk-fat content is commonly used as a diagnostic tool for SARA. A normal milk-fat test in Holstein dairy cows is >4%, so a milk-fat test of <3% can indicate SARA. However, bulk tank testing of milk fat is inappropriate to diagnose SARA at the herd level, so when >4 cows out of 12 and <60 days in milk are suspected to have SARA it can be considered that the herd has a problem. The rapid or abrupt introduction of fresh cows to high-concentrate diets is the most common cause of SARA. Changes in ruminal bacterial populations when exposed to higher concentrate rations require at least about 3 weeks, and it is recommended that concentrate levels increase by no more than 400 g/day during this period to avoid SARA. Ketosis, a prevalent metabolic disorder in dairy cattle, is scrutinized with a focus on its etiological factors and the physiological changes leading to elevated ketone bodies. In total mix ration-fed herds, an increased risk of mastitis and reduced fertility are usually the first clinical signs of ketosis. All dairy cows in early lactation are at risk of ketosis, with most cases occurring in the first 2–4 weeks of lactation. Cows with a body condition score ≥3.75 on a 5-point scale at calving are at a greater risk of ketosis than those with lower body condition scores. The determination of serum or whole blood acetone, acetoacetate, beta-hydroxybutyrate (BHB) concentration, non-esterified fatty acids (NEFA), and liver biopsies is considered the best way to detect and monitor subclinical ketosis, while urine or milk cowside tests can also be used in on-farm monitoring programs. Concentrations >1.0 mmol/L or 1.4 mmol/L blood or serum BHB are considered diagnostic of subclinical ketosis. The standard threshold used for blood is 1.2 mmol/L, which corresponds to thresholds of 100 mcmol/L for milk and 15 mg/dL for urine. Oral administration of propylene glycol (250–400 g, every 24 h for 3–5 days) is the standard and most efficacious treatment, as well as additional therapy with bolus glucose treatment. Hypocalcemia is a disease of adult dairy cows in which acute hypocalcemia causes acute to peracute, afebrile, flaccid paralysis that occurs most commonly at or soon after parturition. Dairy cows are at considerable risk for hypocalcemia at the onset of lactation, when daily calcium excretion suddenly increases from about 10 g to 30 g per day. Cows with hypocalcemia have a more profound decrease in blood calcium concentration—typically below 5.5 mg/dL. The prevention of parturient paresis has been historically approached by feeding cows low-calcium diets during the dry period. Negative calcium balance triggers calcium mobilization before calving and better equips the cow to respond to the massive calcium needs at the onset of lactation. Calcium intake must be limited to <20 g per day for calcium restriction to be effective. The most practical and proven method for monitoring hypocalcemia is by feeding cows an acidogenic diet for ~3 weeks before calving. Throughout the review, emphasis is placed on the importance of early diagnosis and proactive management strategies to mitigate the impact of these metabolic diseases on dairy cattle health and productivity. The comprehensive nature of this paper aims to serve as a valuable resource for veterinarians, researchers, and dairy farmers seeking a deeper understanding of these prevalent metabolic disorders in dairy cattle.

## 1. Introduction

In today’s intensive dairy farming, high demands are placed on the organism of each cow during the production cycle [1,2]. Requirements include the production of large quantities of excellent quality milk and the birth of one calf per cow annually. For example, a cow in peak lactation is expected to reach a production of 30–40 L of milk per day, milk fat content of 3–5%, milk protein content of 3–3.2%, and somatic cell count below 400,000 [3,4,5]. When combined with an ideal production cycle of 305 days of lactation and a dry period of 60 days, along with a reproductive cycle consisting of a service period of 80 days and a pregnancy duration of 285 days, it can be concluded that today’s demands on a living organism, in this case, a dairy breed cow, are very high [6,7].

In this cycle, balanced nutrition is crucial, depending on the specific period of the production cycle. For farmed animals, such as dairy cattle, health and biological functioning are often prioritized. It is well known that during the peripartum period, dairy cows are generally under a state of negative energy balance (NEB), during which they mobilize body fat reserves to provide NEFAs. Despite the action of homeostatic mechanisms to maintain blood parameters within physiologic levels, changes in metabolites and hormones occur as a result of increased metabolic demands in lactating animals. These changes are not necessarily indicative of diseases but make animals physiologically unstable and more susceptible to a number of metabolic diseases at this stage than during other life periods, compromising productivity. In recent years, nutritional strategies have emerged and have been proposed as a key factor to improve the health status and welfare of animals, as well as to enhance productivity in livestock [8,9,10,11]. The most critical period in dairy farming is the transition period [12]. The transition period (the period 3 weeks before and 3 weeks after calving) is when there is a rapid increase in the requirements for substances necessary for milk synthesis. Since milk production rapidly increases from zero to the quantities needed for calf nutrition, the adjustment must be quick, leading to a mismatch between needs and adaptability. Due to these reasons, metabolic disorders often occur at the beginning of lactation [13,14,15].

The colostrum quantity In dairy cows is approximately 10 kg, and by the peak of lactation, the daily amount of milk produced increases to over 40 kg. Milk from cows typically contains an average of 4.1% fat, 3.4% protein, 4.6% lactose, and 0.7% ash. Based on the mentioned data, it can be calculated that a cow at the peak of lactation can secrete around 1600 g of fat, 1300 g of protein, 1800 g of lactose, and 280 g of minerals per day [16]. Given this, the significance of this period cannot be emphasized enough, as the most significant problems with long-term consequences often arise in these 6 weeks. As a rule, care for the health of dairy cows must begin three weeks before the expected calving, or even much earlier [17].

During the transition period, certain physiological changes occur, such as the intensive growth of the fetus, a reduction in the volume of the rumen, the development of the mammary gland for milk synthesis after calving, social changes, and alterations in the environment where the cow resides [18]. In summary, during the transition period, there is up to a 40% reduction in dry matter intake, and the cow’s nutritional needs are dramatically increased (up to three times for glucose and two times for amino acids) [13]. There is a deficiency of vitamins A and E, leading to a NEB [19]. Potential reasons that can explain some of the results obtained by Buonaiuto et al. [20] may be related to the status of negative NEB that commonly occurs in the periparturient period. Plaizier et al. [21] reported that, in addition to NEB, cows can also experience a negative nitrogen balance in the first days after calving. In this phase, dairy cows cannot fulfill the energy deficit by increasing their feed intake [22]. Straczek et al. [23] reported that lactating dairy cows are characterized by high plasma levels of leptin, an anorectic hormone, directly related to a high loss of body condition caused by intensive lactogenesis. Therefore, cows are forced to mobilize body reserves, like fat and muscle tissue. During early lactation, a cow can lose around 20 kg of muscular tissue and between 8 to 57 kg of body fat [24]. Mammals are physiologically unstable and susceptible to a number of metabolic diseases during the peripartum period, compromising productivity [25,26,27]. During the peripartum period, dairy cows face a dysfunctional immune system and an increased inflammatory state due to the modulation of pathways related to metabolism, immune status, and the endocrine system [28].

The aim of this review is to provide a brief overview of the most important metabolic disorders in dairy cows during this critical transitional period. Focus will be placed on disorders such as SARA, ketosis, and hypocalcemia. These diseases represent a growing problem in the dairy industry, even on well-managed farms, as silent, covert, or very dramatic cases of these diseases can directly or indirectly cause financial losses to the farm owner. This is because they directly impact the quantity and quality of the obtained milk, the animals’ production rate, and the profitability of dairy farming itself.

## 2. Subacute Ruminal Acidosis

**Figure 1:**
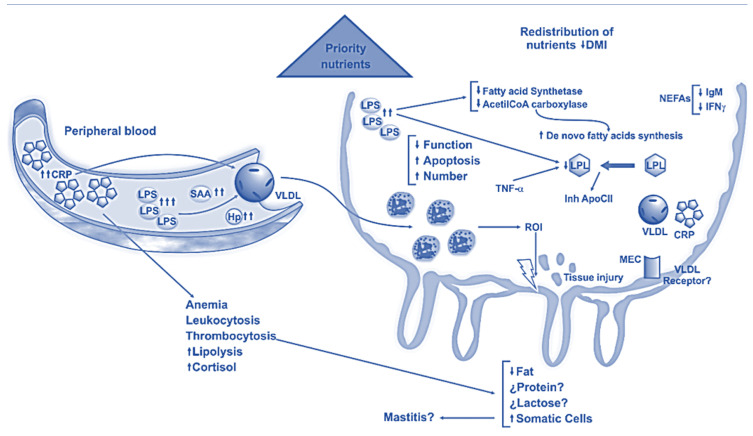
Subacute ruminal acidosis effects on mammary gland [33].

SARA is a digestive metabolic disorder that is increasingly prevalent in dairy cow herds [29,30]. In SARA, there is a decrease in the pH value of the rumen content below the critical point of 5.5 for longer periods, leading to a complex disturbance that significantly disrupts normal metabolic processes [31]. Due to the elevated intake of carbohydrates in the feed, dairy cows release an excessive amount of volatile fatty acids in the rumen, causing the pH value of the rumen content to drop below 5.5 [32]. Milk production decreases (Figure 1), the health of the animals is compromised, and a large number of cattle at the herd level may need to be culled.

Ruminants reflexively stop eating, rumination slows down, and there is mild diarrhea characterized by pasty feces sometimes containing gas bubbles. The greatest risk for the onset of the disease is within 60 days after calving. SARA, as a result of certain feeding programs on dairy farms, tends to occur more frequently as a continuous rather than a transient disorder [34].

### 2.1. Etiology and Pathophysiology

Feeding dairy cows meals with an excessive proportion of carbohydrates causes changes in the composition and quantity of the micro-population in the rumen [35]. With a sudden increase in the quantity of bacteria that release lactic acid through their metabolism, there is a rapid rise in the concentration of lactic acid in the rumen [36]. Lactic acid damages the rumen wall and, in doing so, passively enters the bloodstream, causing changes in the liver, lungs, heart valves, kidneys, and joints. If the increased carbohydrate intake persists for long enough, harmful substances damage the capillary system of the organism, making SARA the most significant predisposing factor for the development of laminitis [37,38]. Nowadays, the loss of body condition due to, e.g., ketosis is considered to be much more related to laminitis or claw-horn disruption [39].

The epithelium of the rumen mucosa is not protected by a mucous layer, making it very sensitive to the action of acids. An increase in the proportion of organic acids, especially lactic acid, lowers the pH value of the rumen content and causes reduced rumen motility [40]. Even under physiological conditions, when the pH value of the rumen content drops to less than 5.5, the release of the hormone secretin begins in the small intestine, slowing down the motility of the rumen. Increasing the frequency of feeding, from twice a day to six times a day, can reduce variations in rumen pH after feeding, but it can also lead to increased feed intake and ultimately cause a decrease in rumen pH content [41]. This mechanism is harmful because retaining acidic content in the rumen allows the absorption of larger amounts of lactic acid into the bloodstream. After the pH of the rumen content drops to around 5.6, the intake of solid matter noticeably decreases [42]. Prolonged low pH values in the rumen content often cause hyperkeratosis, ruminitis, erosions, and ulcers on the rumen epithelium [43]. In addition to ulcers on the rumen epithelium, nowadays, ulcers in the abomasum are more frequently seen than in the rumen. Abomasal ulcers affect mature cattle and calves and have several different manifestations [44]. Common clinical signs include anorexia, bruxism, abdominal pain, occult blood in the feces, and tachycardia. Except for lymphosarcoma of the abomasum and the erosions of the abomasal mucosa that develop in viral diseases such as bovine viral diarrhea, bovine leukemia virus, and bovine malignant catarrhal fever, the causes of abomasal ulceration are not well understood. Although abomasal ulcers can occur at any time during lactation, they are common in high-producing, mature dairy cows within the first 6 weeks after parturition [45].

### 2.2. Clinical Presentation

A herd of dairy cows experiencing SARA often does not show clear clinical signs. The greatest risk for the onset of the disorder is within 60 days after calving. In most animals, only transient rumen hypotonia is observed, accompanied by a slightly reduced appetite and less frequent rumination [38]. A more evident clinical sign of subacute ruminal acidosis, noticeable at the herd level, is reduced feed intake (when the pH value of the rumen content drops below 5.5), decreased milk production, reduced milk fat content in the milked milk, poor body condition of cattle despite a nutritionally well-balanced diet, sudden diarrhea, and the onset of laminitis. The number of culled cows may increase, and cow mortality may rise without a clear cause of death. Spontaneous nosebleeds due to the development of the *venae cavae caudalis* syndrome are also possible [37].

When the pH value of the rumen content drops below 5.5, ruminants reflexively stop eating, rumination slows down, and mild diarrhea characterized by pasty feces containing gas bubbles may occur. The period of reduced feed intake usually lasts for several days. Cattle resume optimal feed intake when the rumen micro-population adapts to the increased carbohydrate intake, and the pH value of the rumen content rises to above 5.5. Laminitis is often described as one of the symptoms of subclinical ruminal acidosis. It can be acute, subacute, or chronic [38]. Recognizing the initial signs of subclinical ruminal acidosis and subclinical laminitis is a significant challenge for the dairy industry. The cause-and-effect relationship between acidosis and laminitis is likely a change in the hemodynamics of peripheral capillary blood flow [37]. The most common signs of subclinical laminitis are bleeding and a yellowish discoloration of the soles. Other clinical signs may develop—such as double sole and erosions of the sole, as well as concave distortion and folding of the dorsal wall of the hoof—with sensitive walking, and so short steps are needed [46].

### 2.3. Diagnosis

During the diagnosis of SARA, health disorders that may arise due to spoiled silage, poor diet composition, and errors in feeding table setup must be ruled out. Because of the unclear symptoms that appear in each animal affected by SARA, diagnostic procedures are employed to assess parameters studied at the herd level.

Determining the pH value of rumen contents is the most commonly used method in diagnosing SARA in dairy cow herds [47]. Animals in the first month of lactation are selected for sampling, and the sample must include at least 12 sampled cattle. Samples are collected 2 to 4 h after being fed fresh meals. Rumen contents are sampled either by ruminocentesis or by using a rumen probe. Measuring the pH value of the sampled rumen contents with indicator paper, which has a pH measurement range of 2 to 12, yields satisfactory results [48]. If the pH value of rumen contents is found to be less than 5.5 in over 25% of cattle in the selected sample, there is considered to be a high risk of SARA development. In a sample of 12 cows, having 2, 3, or 4 cows positive (with a lower pH of rumen contents below 5.5) would indicate a critical situation in that herd. If 4 or more cows out of the 12 sampled are positive, meaning they have a pH lower than 5.5, the herd is considered positive for SARA. The procedure for measuring the pH value of rumen contents should be applied in conjunction with other diagnostic methods, such as evaluating the quality of the cattle’s diet, assessing herd management, and identifying other health problems at the herd level [49]. A decrease in the milk fat percentage has been proven to be an unreliable indicator in diagnosing subacute ruminal acidosis [50].

### 2.4. Prevention of SARA

Clinical signs of SARA manifest only after a certain period from the commencement of improper feeding of cows [51]. Therefore, preventive measures should be primarily implemented in dairy cow herds to prevent the occurrence of SARA. After confirming the presence of SARA in the herd, and before taking any suppression measures, it is essential to identify the cause of its occurrence [37]. The causes are typically grouped into three categories: excessive carbohydrate intake in the diet, inadequate rumen buffering, and poorly conducted rumen adaptation to diets with increased carbohydrate content [52].

During the early lactation period, prevention is of utmost importance. It is crucial to ensure a gradual increase in the proportion of carbohydrates in the diet during the first six weeks after calving. The best prevention of SARA is achieved by aligning the increase in carbohydrate content in the diet with an increase in dry matter intake. Special feeding patterns have been developed, prescribing acceptable carbohydrate proportions when formulating diets for cows in the first six weeks of lactation, and maintaining raw fiber intake without compromising energy levels to prevent ketosis [53]. Another risk moment is when cows obtain the maximal carbohydrate intake. It is assumed that the weekly increase in carbohydrate intake should be only 0.9 to 1.6 kg. Scientists recommend adding various preparations to prevent subacute ruminal acidosis. Adding monensin to the feed redirects the metabolism of volatile fatty acids and increases propionate production, which further stimulates gluconeogenesis. Monensin also has a favorable effect on preventing ketosis as it increases milk production, although it simultaneously reduces the percentage of milk fat [54].

Adding lactate to the diet in the late dry period can help with a faster adaptation of the micro-population to diets with increased carbohydrate content [55]. To facilitate adaptation to the increased lactate content, bacteria that are direct consumers of lactate can be applied to the rumen, thus temporarily reducing the risk of a decrease in the pH value of rumen contents.

Preventing SARA also involves assessing the actual proportions of all ingredients in the diet. Determining the actual values of the consumed diet is possible only through a careful evaluation of all steps in the ingredient processing, preparation, and delivery of the finished diet to the feeding table. Careful and proper sampling of a composite sample and analyzing all ingredients can help us to uncover hidden errors in the composition of the diet delivered to the cattle. It is considered that herds with an identified increased intake of dry matter in the diet have a significantly higher risk of developing SARA [56]. In such herds, it is urgently necessary to reduce the proportion of carbohydrates in the diets. The proper representation of all feed ingredients in any part of the diet is crucial in the feed preparation process.

The delivery of feed to the feeding table and the possibility of free access for each animal to the offered feed are often the most underestimated aspects of herd management. Dairy cow herds are most often fed ad libitum to achieve the highest nutrient intake in each cow, aiming to increase milk production [57]. Introducing dietary restrictions during the period of the highest risk of SARA is believed to reduce the occurrence of this disorder [58].

Diets with overly fine particles of raw fiber increase the risk of SARA [59]. However, diets with excessively long pieces of raw fiber can also increase the risk of SARA as they allow for easier sorting of feed at the feeding table. Dominant cows, which are usually the first to access the feeding table, tend to sort the feed, consuming portions with the highest energy content and insufficient levels of rough fiber, making them more susceptible to the development of SARA.

## 3. Ketosis

Ketosis is a common metabolic disorder in dairy cows that occurs 2–4 weeks after calving, characterized by an increased concentration of ketone bodies (BHB) in blood, milk, and urine [60]. Cows with clinical ketosis exhibit symptoms such as anorexia, abnormal licking and chewing, rapid weight loss, and reduced milk production [61,62]. Ketones are byproducts of the conversion of fats into carbohydrates, and ruminants use them as an energy source to a limited extent under physiological conditions [63]. Crucially, reducing the severity and duration of NEB is vital for preventing ketosis [64].

### 3.1. Etiology and Pathophysiology

Favorable factors for cows’ metabolic predisposition to ketosis include their milk constitution, while immediate errors in nutrition and the failure of neurohormonal regulation of metabolism contribute to the problem. Cows are physiologically inclined to a shortage of carbohydrates, specifically glucose, making the process of gluconeogenesis from other sources (propionic acid, glycerol, and proteins) crucial for maintaining metabolic balance. An excess of concentrates in the diet disrupts the physiological balance of volatile fatty acids in the rumen, favoring ketogenic acids (acetic and butyric acid) at the expense of propionic acid. At the beginning of lactation, cows face sudden and drastically increased energy demands [65]. The increase in postpartum feed intake lags behind the energy needs during lactation, leading to a NEB [66]. Fats are mobilized from body reserves in the form of NEFA to meet the energy demand. NEFA travels to the liver where it is utilized in three ways: complete oxidation into energy, incomplete oxidation into ketone bodies, or re-esterification into fatty acids [67]. In early lactation, homeorhesis is a leading physiological process [68].

Additionally, conditionally pathogenic microorganisms causing continuously present, common, and sometimes fatal infections (mostly in the digestive and respiratory systems, mammary glands, and reproductive system) can be attributed to the mentioned etiological factors. Due to the lack of oxaloacetic acid, there is increased fat breakdown in the liver, resulting in abundant ketone formation [68]. Ketones accumulate in the blood (ketonemia) and tissues and are excreted in urine (ketonuria) [69].

The liver can compensate for the metabolic changes that occur, and decompensation begins when glycogen and biocatalyst reserves are depleted, leading to hepatic fatty degeneration and later cirrhosis. Among the ketone byproducts, acetoacetic acid is the most important, playing a crucial role in the organism’s intoxication [70]. Due to the accumulation of other acidic degradation products, changes in substances occur, resulting in metabolic acidosis and dehydration, further complicating the already disturbed overall condition of the animal [71].

Type 1 ketosis is spontaneous ketosis occurring in undernourished cows and is also known as thin cow syndrome. It typically manifests in cows 2 to 4 weeks after calving [63]. Named type 1 due to its similarity to type 1 diabetes mellitus, both conditions exhibit reduced insulin concentration in the blood, although the causes differ. In diabetes, insulin concentration decreases due to pancreatic hormone secretion disorders, while in ketosis, it is low due to chronic hypoglycemia [72]. Cows with type 1 ketosis can produce glucose from precursors (rumen propionate and amino acids from the small intestine). The limiting factor is the supply of glucose precursors. Under these conditions, ketone concentration in the blood becomes very high, and glucose concentration becomes very low [73].

Type 2 ketosis, also known as fat cow syndrome, encompasses obese cows and those experiencing NEB and fat mobilization just before calving. The fundamental change in type 2 ketosis is fatty degeneration of the liver, clinically evident after calving. Obese cows are at the greatest risk because they are prone to reduced feed intake during calving [74]. Excessive lipolysis is evident in significantly increased NEFA concentrations and more massive liver triglyceride accumulation. During intensive gluconeogenesis, large amounts of NEFA from the serum are redirected to the liver, where they are synthesized into ketone bodies. Ketone bodies consist of 70% BHB, 28% acetoacetate, and 2% acetone [75]. The predominant ketone body is dependent on the stage of lactation [76]. Fatty degenerated livers have reduced gluconeogenic potential, and disease signs in cows appear before or within the first weeks after calving. Type 2 ketosis is metabolically similar to type 2 diabetes, with both conditions exhibiting elevated insulin and glucose concentrations in the blood (transiently in cows with ketosis), interpreted as tissue insulin resistance [77]. Obesity seems to play a significant role in tissue insulin resistance. In obese cows, fat mobilization worsens existing liver fatty degeneration, promotes ketone formation, and reduces appetite [63]. Ketone concentration is elevated but is still lower than in type 1 ketosis. Fatty degenerated hepatocytes have reduced gluconeogenic potential, and the liver’s immune response is compromised [78]. Often, such cows succumb to infections of different origin.

Type 3 ketosis is caused by an excess of butyric acid in feed (butyric acid silage ketosis). Some herds constantly face ketosis issues due to being fed ketotic silage. If bacteria of the *Clostridium* sp. develop in silage due to moisture and other favorable conditions, carbohydrates are metabolized into butyric instead of lactic acid [79]. Silage with clostridial microflora has a specific rancid smell of butyric acid. To confirm significant amounts of butyric acid, a laboratory analysis of the suspicious silage sample is required for clostridial fermentation [80]. Various studies have shown that a daily dose of 50–100 g of butyric acid can cause subclinical ketosis, while a dose exceeding 200 g leads to the development of clinically manifested ketosis [81,82]. Cows primarily use butyric acid as an energy source for the rumen muscle. Approximately 75% of the remaining butyric acid is converted into BHB, one of the direct causes of ketosis. The liver can transform the produced BHB into acetoacetic acid or reverse the process [83]. The daily dose of butyric acid for dairy cattle should be less than 50 g per cow. Aerating silage before feeding cows can reduce butyric acid content by 50%, making it more suitable for feeding [84].

### 3.2. Clinical Presentation

The clinical presentation of ketosis develops in two phases: the subclinical or latent phase, and the clinical, clearly expressed phase. Subclinical ketosis in dairy cows is the presence of ketone bodies in circulation without the presence of clinical signs of ketosis [79].

Subclinical ketosis (latent phase) manifests as nonspecific general disturbances in metabolic balance. It includes unbalanced milk production, a tendency to lose weight despite appetite and normal rumen function, and occasional moderate ketonuria. A cow with developed subclinical ketosis may not always be thin (for example, in the case of fat cow syndrome). Unbalanced milk production is characterized by significant daily variations in milk quantity without a visible cause [85]. Biochemically, subclinical ketosis is marked by hypoglycemia and ketonemia [86]. Subclinical ketosis remains latent until the increase in ketone concentration exceeds the blood glucose concentration. Due to its latent course and significant production losses, subclinical ketosis has considerable practical and economic importance. The prevalence of subclinical ketosis in early lactation dairy cows ranges from 7.5% to 14%. Cows with subclinical ketosis have a 4.9 times higher chance of developing metritis, a 6.1 times higher chance of developing displaced abomasum, and a 1.98 times higher chance of developing hoof diseases [87].

The clinically manifested phase occurs when the concentration of ketones in the blood exceeds the concentration of glucose, but it can also manifest earlier if hormonal regulation fails. Initially, general symptoms of disrupted energy balance and indigestion syndrome predominate in the clinical presentation. Consequently, there is rapid weight loss in early lactation, reduced milk production, decreased fertility, higher veterinary service costs, and ultimately a greater number of culled cows. Body temperature can be normal, but it may also be reduced, mainly due to decreased oxidative processes in the body. Bradycardia and bradypnea may occur [88]. Most animals are lethargic, have a reduced or variable appetite, and may exhibit abnormal behavior.

Rumen function is disturbed, leading to acidosis or obstruction. Animals may emit a smell of acetone, especially in their breath and milk, which is also bitter in taste. In severe cases, the entire herd may have an acetone odor [89]. These symptoms result from an increase in ketone concentration in the blood, rumen contents, and urine. The total concentration of ketones in the blood can rise by 5 to 10 times compared to the physiological level, and, in urine, it can increase by 10 to 100 times [79]. If ketone bodies are found in the urine on the 21st day of the disease, and preventive measures and treatment procedures have been taken before that, such a cow should be culled [75]. In more severe and advanced cases, signs of metabolic acidosis, dehydration, and central nervous system disorders may accompany ketosis. Acidosis occurs due to the accumulation of acidic by-products of metabolism and reduced alkaline reserves. Sodium ion loss leads to water loss from the body [63]. Dehydration is manifested by hemoconcentration and relative hyperproteinemia. Skin turgor is reduced, and the eyes are sunken. Central nervous system symptoms arise from reduced oxidative processes in the nervous system (due to carbohydrate deficiency) and the toxic effects of metabolic by-products, especially acetoacetic acid. In acute cases, specific symptoms of nervous system diseases occur, such as excitement, paresis, paresthesia, and hyperesthesia. Animals chew empty air, grind their teeth, bellow, have a wild look, and saliva drips from their mouths. A form of ketosis similar to puerperal paresis occurs after calving, and the symptoms resemble puerperal paresis, except that the pupillary reflex is preserved, and calcium therapy is ineffective [90]. In advanced cases, animals fall into a stupor, and, eventually, into a coma. Symptoms originating from the liver are important for the prognosis and outcome of the disease. In milder cases of ketosis, clinical and biochemical signs of liver disease are usually not found [91]. In severe and advanced cases, and if the process is chronic, an increased, moderately sensitive liver can be observed during percussion and palpation (in the area behind the right last rib) [92]. Histologically, there is fatty degeneration (steatosis) of the liver, and in advanced cases, cirrhosis may occur.

### 3.3. Diagnosis

In subclinical cases, ketotic conditions can only be detected through the systematic testing of urine and milk for ketone bodies. Animals in a herd or flock are selected for testing based on clinical examination and milking data. Unbalanced milking during the full lactation period most commonly indicates a disturbance in metabolic balance [93].

The gold standard in the laboratory diagnosis of subclinical ketosis is the concentration of BHB in the blood and the liver biopsy [94]. Subclinical ketosis is considered when the concentration of BHB in the blood is higher than 0.85 mmol/L. Research has shown a prevalence of ketosis in herds of around 15%, with a critical level set at 10% [95]. The concentration of BHB in the blood increases after feeding, and sample collection should follow at intervals of 4 to 5 h after the first meal. The sample should consist of 12 sampled cows from a group of 50 cows. If one or two samples are positive, indicating elevated BHB values, the herd’s condition is critical. A herd sample in which two or more cows in a group of twelve are positive is declared positive for ketosis [60].

The concentration of acetoacetic acid can also be determined in the blood (possibly in milk and urine) [96]. Subclinical ketosis is considered when the concentration of acetoacetic acid is higher than 0.36 mmol/L, while clinically manifested ketosis occurs when the concentration of acetoacetic acid exceeds 0.5 mmol/L [97]. Acetoacetic acid is not the best laboratory parameter for diagnosing ketosis because it is unstable and rapidly breaks down into acetone and carbon dioxide [98].

The concentration of ketone bodies in milk is approximately 50% lower than in blood, and the concentration in urine is several times higher than that in blood [99]. An increase in the concentration of ketone bodies in the blood accompanies a decrease in blood glucose concentration. In cattle, the normal concentration of blood glucose ranges from 2.3 to 4.1 mmol/L, and if it is less than 2.3 mmol/L or lower, the animal is considered to be suffering from ketosis [100]. Another valuable indicator is NEFA. The threshold value for NEFA is more than 0.400 mEq/L in cows 2 to 14 days before calving and 4 to 5 h after the first meal of the day [101]. It is crucial to store the samples properly, keeping them cool or even frozen from the collection to the laboratory delivery.

Ketone bodies in urine can be detected using sodium nitroprusside, the legal test [102]. A positive reaction is indicated by a color change in urine, ranging from pink (+) to pink/purple (++) or dark purple (+++), depending on the ketone concentration. Today, numerous rapid methods for detecting ketones in milk and urine (known as dipstick tests) exist, allowing for the straightforward detection of acetone and acetoacetic acid in milk and urine [94].

### 3.4. Treatment and Prevention of Ketosis

Subclinical ketosis leads to a threefold higher culling rate of cows within the first 30 days of lactation compared to cows that are negative for ketosis. Additionally, small-scale producers often overestimate the incidence of clinical ketosis on their farms [82].

It is possible to administer 25% glucose in a quantity of 20 L as a slow infusion over 24 h. Oral administration of metabolic carbohydrate precursors is indicated, primarily propylene glycol (225 g, 2×/day for two days, then 100 g, 1×/day for two days) and glycerol (500 g, 2×/day for 10 days). Cows with subclinical ketosis treated with oral propylene glycol had a 1.3 times higher chance of conception at the first subsequent insemination [103].

Positive metabolic effects are achieved through the use of low doses of long-acting insulin (in combination with glucose infusions or glucocorticoid administration). Insulin reduces the mobilization of fat from tissue reserves, promotes glucose entry into cells, and stimulates glycolysis in the liver. Human ultra-long-acting insulin is most commonly administered at a dose of 0.25 IU/kg body weight subcutaneously every 24 to 48 h. In the treatment and prophylaxis of ketosis, additional therapy with vitamins and minerals is used [104]. Thus, ketotic animals are administered vitamin B12, cobalt, niacin (6 g orally), and nicotinamide. If there are symptoms of central nervous system involvement, the administration of chloral hydrate is indicated. In addition to its sedative effect, chloral hydrate has been shown to increase the concentration of propionic acid in the rumen. The initial dose is 30 g orally, and treatment continues with a dose of 7 g, 2×/day for several days. The best results in the treatment and prophylaxis of ketosis in high-yielding dairy cows have been achieved with the use of the ionophore monensin. Monensin acts on the rumen micro-population, increasing the availability of propionic acid and thus suppressing fat mobilization and ketone body formation [105]. The use of monensin significantly reduced the incidence and duration of subclinical ketosis by as much as 50% by measuring BHB concentration in the blood [106]. The prognostic condition of this disease is unfavorable during periods of depression, leanness, and if cows do not respond to therapy and dietary changes.

Ketosis prevention is primarily based on balanced nutrition, achieving a positive energy balance, and a proper lactation and dry period regimen. On average, feed rations are increased from the fourth to the sixth week before calving, reaching their maximum approximately two weeks before calving [75]. This does not mean, of course, that cows should be fattened during this period. Good milking cows should be fed during the dry period as if they were producing 5 L of milk daily, in addition to a modest diet. To prevent carbohydrate deficiency and thus the onset of ketosis, after calving and the onset of the first lactation, cows can be given a carbohydrate precursor such as propylene glycol with their meal. The propionate from propylene glycol is used for gluconeogenesis and stimulates insulin secretion [18].

## 4. Hypocalcemia

Afebrile postparturient hypocalcemia is a condition in adult, high-yielding cows, and it most commonly occurs immediately after calving and at the beginning of lactation [107]. It manifests as sudden paralysis and loss of consciousness, and, if left untreated, can lead to a fatal outcome [108].

### 4.1. Etiology and Pathophysiology

Hypocalcemia occurs due to the sudden production of large quantities of milk and the acute depletion of ionized calcium reserves in the serum. Animals attempt to meet increased calcium demands through enhanced absorption from the digestive system and mobilization from bone stores [109]. Subclinical hypocalcemia, where the drop in calcium levels is not as pronounced, affects 50% of lactating cows. If animals are adequately supplemented with minerals, the disease risk decreases from 25 to 15%. Older cows, from the third to the seventh lactation, are more likely to be affected, and the disease is relatively rare in first-calvers [110].

For the development of the disease in dairy cows, nutrition in the last 4 weeks of pregnancy is crucial. It has been proven that the disease is more common if cows receive a calcium-rich diet before calving. Consequently, these cows are unable to immediately utilize calcium from bones or actively absorb calcium from the digestive system during calving. Instead, they need several days to activate these mechanisms, making them highly susceptible to puerperal paresis during this period [111].

The concentration of calcium in the blood is regulated by parathyroid hormone (PTH) and 1,25-dihydroxyvitamin D_3_, produced in response to hypocalcemia to increase blood calcium levels [112]. If calcium levels are only moderately reduced, PTH stimulates renal calcium absorption from the glomerular filtrate, and calcium levels soon return to normal values. However, if calcium levels are too low, PTH continues to stimulate calcium resorption from bones [113]. After calving, a cow produces up to 10 L of colostrum, and since colostrum production begins immediately after calving and lasts 5 to 12 days, the calcium requirements are high [114]. A cow loses 23 g of calcium per liter of milk, which is approximately nine times more than the amount of calcium in the blood [115]. During the dry period, these mechanisms are inactive, and all cows in the first few days after calving are susceptible to hypocalcemia. They are unable to immediately utilize calcium from bones or actively transfer calcium from the digestive system within the first 24 to 48 h, after which resorption significantly increases. For this system to function optimally, the crucial prerequisite is an optimal blood pH of 7.4. If blood pH becomes higher than 7.5, metabolic alkalosis occurs, predisposing cows to hypocalcemia [116]. The cause of metabolic alkalosis is large amounts of sodium and potassium in the cows’ dry period diet [117]. Potassium is a more significant issue because all forages contain large amounts of potassium. When blood pH becomes alkaline, the structure of PTH receptors changes, and PTH does not act as efficiently as it should. Most nutritionists aim to maintain a dietary cation/anion difference (DCAD) [(Na^+^ + K^+^) − (Cl^−^ + S^−^)] of around −50 mEq/kg preventively [118,119].

### 4.2. Clinical Presentation

**Figure 2:**
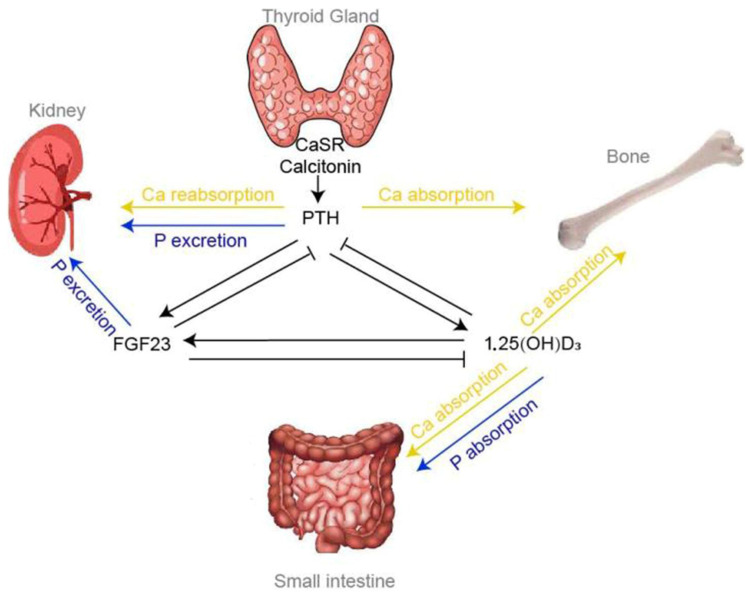
Endocrine control of extracellular calcium homeostasis [122].

Clinical signs manifest in three stages. In the first stage, there is nerve and muscle hypersensitivity, excitement, muscle tremors, anorexia, and ataxia. The animal refuses to move or eat, but its temperature remains normal. This condition can last for hours. Animal owners often overlook the first stage, leading to a worsening condition in affected cows [116]. Subclinical hypocalcemia results in higher costs because it affects a much larger percentage of animals in the herd [115]. For example, if a herd of 2000 cows has a 2% annual incidence of milk fever (Figure 2), and each case costs 334 US dollars [120], the annual loss amounts to approximately 12,000 dollars. Recent studies show that hypocalcemia during the calving period is associated with a loss in milk yield and an increased risk of abomasal displacement [121].

The second stage is the prodromal stage, characterized by extreme exhaustion and a loss of strength. The animal cannot stand and lies in a sternal position. Tetany, present in the first phase, is replaced by prolonged lying down and paresis. Depression, anorexia, dry nose, subnormal body temperature (36.5 to 38 °C), and cold extremities become noticeable. Arterial pulse is weak, heartbeats are barely audible, and the frequency is moderate (80/min). The paralysis of smooth muscles leads to gastrointestinal hypotonia and abomasal atony, which can manifest as bloat [115,123,124,125]. Simultaneously, the ability to urinate is lost. Due to uterine inertia, a retained placenta occurs. A noticeable decline in milk yield of 14% occurs, and cows are more prone to various diseases: ketosis, retained placenta, and abomasal displacement [126].

In the third stage, hypocalcemia progresses; the animal loses consciousness and falls into a coma. Cows lie in a lateral position, and bedsores may develop. Advanced-stage heart depression is evident, with an irregular and almost imperceptible pulse. Breathing is shallow and reduced. Cows in this stage of complete collapse do not survive longer than a few hours [127].

### 4.3. Diagnosis

Before treatment, it is essential to take a blood sample to confirm the diagnosis by finding a decreased calcium level in the serum. Cows with serum calcium values less than 8.0 mg/dL are considered hypocalcemic. The blood sample is best taken 12 to 24 h after calving, with a minimum of 12 samples [128]. If three to five samples are positive, meaning they have a serum calcium value less than 8.0 mg/dL, it is considered a critical level. A sample in which six or more cows in a group of twelve are positive is declared positive, meaning that the herd is hypocalcemic [129].

For DCAD verification, urine pH has proven to be a good diagnostic method since blood pH should be around 7.0. The minimum number of urine samples for diagnostic purposes is eight cows, and testing would be advisable every week, if not more frequently. The procedure is straightforward as a regular pH indicator paper is sufficient [130].

### 4.4. Treatment and Prevention of Hypocalcemia

Treatment is carried out by the intravenous administration of calcium borogluconate at a dose of 1 g of calcium per 45 kg of the animal’s body weight. In the treatment of large cows with high milk production, an additional bottle of the drug can be administered subcutaneously [131]. As calcium is cardiotoxic, the drug should be administered extremely slowly (over 10 to 20 min), carefully monitoring the heart’s activity through auscultation [132]. If bradycardia or arrhythmia is observed, treatment should be stopped, and it can be resumed very slowly only when the heart’s activity normalizes.

Intravenous calcium administration is not recommended for treating hypocalcemic cows that are still standing. Treatment with intravenous calcium administration rapidly increases the calcium level in the blood, which can be potentially dangerous [132]. Extremely high levels of calcium in the blood can cause fatal cardiac complications and hinder calcium mobilization in cows at critical moments [126]. Since the subcutaneously administered drug is more challenging to be absorbed due to weakened peripheral absorption, it should not be used as the sole choice. Subcutaneously administered calcium allows the return of serum calcium within physiological limits within 6 h of administration [131]. Oral calcium supplementation is the best choice for hypocalcemic cows that are still standing [133].

Cows that respond to intravenous calcium treatment, once they are conscious and able to swallow, should also be given oral calcium supplementation 12 h after treatment [134].

Prevention is based on feeding cows with low calcium content, especially high-producing cows during the dry period, to stimulate intestinal and bone resorption. Reducing potassium content in the diet during the dry period leads to a decrease in blood pH, thereby promoting calcium resorption [135]. As a preventive measure, 8 million IU of vitamin D and oral calcium supplements (150 g) can be administered eight days before calving, on the day of calving, and on the day after calving. Magnesium also plays a crucial role in calcium homeostasis during the calving period. An intake of 40 to 50 g of magnesium (approximately 0.30–0.45% dry matter in the diet) is also recommended [136].

## 5. Conclusions

This comprehensive review underscores the critical importance of understanding and addressing metabolic diseases, particularly subacute ruminal acidosis (SARA), ketosis, and hypocalcemia, in dairy cattle. By exploring the intricate details of etiology, pathophysiology, clinical presentations, diagnosis, and strategic management, this paper provides a holistic perspective for veterinary practitioners, researchers, and dairy farmers.

The multifaceted nature of these metabolic disorders necessitates a nuanced approach to both diagnosis and treatment. Early detection emerges as a pivotal factor, enabling timely interventions that can significantly impact the course of these diseases. Effective diagnostic tools and protocols are crucial for accurate identification, allowing for tailored treatment strategies.

Moreover, the emphasis on preventative measures cannot be overstated. Nutritional management, balanced diets, and proactive supplementation play pivotal roles in averting the onset of these metabolic diseases. This review serves as a valuable repository of knowledge, equipping stakeholders in the dairy industry with insights to enhance animal welfare, optimize productivity, and reduce economic losses associated with these prevalent disorders. As research advances, ongoing efforts in refining preventive and therapeutic approaches remain essential for ensuring the health and well-being of dairy cattle worldwide.

## Data Availability

Data are provided within the text.

## References

[1] Britt J.H., Cushman R.A., Dechow C.D., Dobson H., Humblot P., Hutjens M.F., Jones G.A., Ruegg P.S., Sheldon I.M., Stevenson J.S. (2018). Invited Review: Learning from the Future—A Vision for Dairy Farms and Cows in 2067. J. Dairy Sci..

[2] Vries A.D., Marcondes M.I. (2020). Review: Overview of Factors Affecting Productive Lifespan of Dairy Cows. Animal.

[3] Pulina G., Tondo A., Danieli P.P., Primi R., Matteo Crovetto G., Fantini A., Macciotta N.P.P., Atzori A.S. (2020). How to Manage Cows Yielding 20,000 Kg of Milk: Technical Challenges and Environmental Implications. Ital. J. Anim. Sci..

[4] Leduc A., Souchet S., Gelé M., Le Provost F., Boutinaud M. (2021). Effect of Feed Restriction on Dairy Cow Milk Production: A Review. J. Anim. Sci..

[5] Doran M.J., Mulligan F.J., Lynch M.B., Fahey A.G., Rajauria G., Brady E.L., Pierce K.M. (2022). Effects of Concentrate Supplementation and Genotype on Milk Production and Nitrogen Utilisation Efficiency in Late-Lactation, Spring-Calving Grazing Dairy Cows. Livest. Sci..

[6] Maltz E. (2020). Individual Dairy Cow Management: Achievements, Obstacles and Prospects. J. Dairy Res..

[7] Davis T.C., White R.R. (2020). Breeding Animals to Feed People: The Many Roles of Animal Reproduction in Ensuring Global Food Security. Theriogenology.

[8] Abbate J.M., Macrì F., Capparucci F., Iaria C., Briguglio G., Cicero L., Salvo A., Arfuso F., Ieni A., Piccione G. (2020). Administration of Protein Hydrolysates from Anchovy (Engraulis Encrasicolus) Waste for Twelve Weeks Decreases Metabolic Dysfunction-Associated Fatty Liver Disease Severity in ApoE^−/−^Mice. Animals.

[9] Piccione G., Arfuso F., Fazio F., Bazzano M., Giannetto C. (2014). Serum Lipid Modification Related to Exercise and Polyunsaturated Fatty Acid Supplementation in Jumpers and Thoroughbred Horses. J. Equine Vet. Sci..

[10] Armato L., Gianesella M., Morgante M., Fiore E., Rizzo M., Giudice E., Piccione G. (2016). Rumen Volatile Fatty Acids × Dietary Supplementation with Live Yeast and Yeast Cell Wall in Feedlot Beef Cattle. Acta Agric. Scand. Sect. A—Anim. Sci..

[11] Monteverde V., Congiu F., Vazzana I., Dara S., Di Pietro S., Piccione G. (2017). Serum Lipid Profile Modification Related to Polyunsaturated Fatty Acid Supplementation in Thoroughbred Horses. J. Appl. Anim. Res..

[12] Lopreiato V., Mezzetti M., Cattaneo L., Ferronato G., Minuti A., Trevisi E. (2020). Role of Nutraceuticals during the Transition Period of Dairy Cows: A Review. J. Anim. Sci. Biotechnol..

[13] Mezzetti M., Cattaneo L., Passamonti M.M., Lopreiato V., Minuti A., Trevisi E. (2021). The Transition Period Updated: A Review of the New Insights into the Adaptation of Dairy Cows to the New Lactation. Dairy.

[14] Caixeta L.S., Omontese B.O. (2021). Monitoring and Improving the Metabolic Health of Dairy Cows during the Transition Period. Animals.

[15] Pascottini O.B., Leroy J.L.M.R., Opsomer G. (2020). Metabolic Stress in the Transition Period of Dairy Cows: Focusing on the Prepartum Period. Animals.

[16] Borchardt S., Sutter F., Heuwieser W., Venjakob P. (2022). Management-Related Factors in Dry Cows and Their Associations with Colostrum Quantity and Quality on a Large Commercial Dairy Farm. J. Dairy Sci..

[17] Roche J.R., Meier S., Heiser A., Mitchell M.D., Walker C.G., Crookenden M.A., Riboni M.V., Loor J.J., Kay J.K. (2015). Effects of Precalving Body Condition Score and Prepartum Feeding Level on Production, Reproduction, and Health Parameters in Pasture-Based Transition Dairy Cows. J. Dairy Sci..

[18] Ingvartsen K.L. (2006). Feeding- and Management-Related Diseases in the Transition Cow: Physiological Adaptations around Calving and Strategies to Reduce Feeding-Related Diseases. Anim. Feed Sci. Technol..

[19] Tamminga S. (2006). The Effect of the Supply of Rumen Degradable Protein and Metabolisable Protein on Negative Energy Balance and Fertility in Dairy Cows. Anim. Reprod. Sci..

[20] Buonaiuto G., Lopez-Villalobos N., Costa A., Niero G., Degano L., Mammi L.M.E., Cavallini D., Palmonari A., Formigoni A., Visentin G. (2023). Stayability in Simmental Cattle as Affected by Muscularity and Body Condition Score between Calvings. Front. Vet. Sci..

[21] Plaizier J.C., Martin A., Duffield T., Bagg R., Dick P., McBride B.W. (2000). Effect of a Prepartum Administration of Monensin in a Controlled-Release Capsule on Apparent Digestibilities and Nitrogen Utilization in Transition Dairy Cows. J. Dairy Sci..

[22] Gáspárdy A., Schwartz Z., Zöldág L., Veresegyházy T., Fekete S. (2004). Changes in Daily Energy Amounts of Main Milk Components (Lactose, Protein and Fat) during the Lactation of High-Yielding Dairy Cows. Acta Vet. Hung..

[23] Strączek I., Młynek K., Danielewicz A. (2021). The Capacity of Holstein-Friesian and Simmental Cows to Correct a Negative Energy Balance in Relation to Their Performance Parameters, Course of Lactation, and Selected Milk Components. Animals.

[24] Komaragiri M.V.S., Casper D.P., Erdman R.A. (1998). Factors Affecting Body Tissue Mobilization in Early Lactation Dairy Cows. 2. Effect of Dietary Fat on Mobilization of Body Fat and Protein. J. Dairy Sci..

[25] Bazzano M., Giannetto C., Fazio F., Arfuso F., Giudice E., Piccione G. (2014). Metabolic Profile of Broodmares During Late Pregnancy and Early Post-Partum. Reprod. Domest. Anim..

[26] Fiore E., Arfuso F., Gianesella M., Vecchio D., Morgante M., Mazzotta E., Badon T., Rossi P., Bedin S., Piccione G. (2018). Metabolic and Hormonal Adaptation in Bubalus Bubalis around Calving and Early Lactation. PLoS ONE.

[27] Fiore E., Gianesella M., Arfuso F., Giudice E., Piccione G., Lora M., Stefani A., Morgante M. (2014). Glucose Infusion Response on Some Metabolic Parameters in Dairy Cows during Transition Period. Arch. Anim. Breed..

[28] Arfuso F., Minuti A., Liotta L., Giannetto C., Trevisi E., Piccione G., Lopreiato V. (2023). Stress and Inflammatory Response of Cows and Their Calves during Peripartum and Early Neonatal Period. Theriogenology.

[29] Abdela N. (2016). Sub-Acute Ruminal Acidosis (SARA) and Its Consequence in Dairy Cattle: A Review of Past and Recent Research at Global Prospective. Achiev. Life Sci..

[30] Elmhadi M.E., Ali D.K., Khogali M.K., Wang H. (2022). Subacute Ruminal Acidosis in Dairy Herds: Microbiological and Nutritional Causes, Consequences, and Prevention Strategies. Anim. Nutr..

[31] Zebeli Q., Metzler-Zebeli B.U. (2012). Interplay between Rumen Digestive Disorders and Diet-Induced Inflammation in Dairy Cattle. Res. Vet. Sci..

[32] McCann J.C., Luan S., Cardoso F.C., Derakhshani H., Khafipour E., Loor J.J. (2016). Induction of Subacute Ruminal Acidosis Affects the Ruminal Microbiome and Epithelium. Front. Microbiol..

[33] Gómez L.M., Posada S.L., Olivera M. (2014). Sub-Acute Ruminal Acidosis and Non-Structural Carbohydrates: A Study Model in Nutritional Immunology. CES Med. Vet. Zootec..

[34] Hayton A., Husband J., Vecqueray R. (2012). Nutritional Management of Herd Health. Dairy Herd Health.

[35] Kitkas G.C., Panousis N., Valergakis G.E., Karatzias C. (2017). Subacute Ruminai Acidosis in Dairy Cows. J. Hell. Vet. Med. Soc..

[36] Kitkas G.C., Valergakis G.E., Kritsepi-Konstantinou M., Gelasakis A.I., Arsenos G., Kalaitzakis E., Panousis N. (2019). Effects of Ruminal pH and Subacute Ruminal Acidosis on Milk Yield and Composition of Holstein Cows in Different Stages of Lactation. J. Hell. Vet. Med. Soc..

[37] Voulgarakis N., Athanasiou L., Psalla D., Gougoulis D., Papatsiros V., Christodoulopoulos G. (2024). Ruminal Acidosis Part II: Diagnosis, Prevention and Treatment. J. Hell. Vet. Med. Soc..

[38] Voulgarakis N., Gougoulis D., Psalla D., Papakonstantinou G., Angelidou-Tsifida M., Papatsiros V., Athanasiou L., Christodoulopoulos G. (2023). Ruminal Acidosis Part I: Clinical Manifestations, Epidemiology and Impact of the Disease. J. Hell. Vet. Med. Soc..

[39] Huxley J.N. (2013). Impact of Lameness and Claw Lesions in Cows on Health and Production. Livest. Sci..

[40] Valente T.N.P., Sampaio C.B., Lima E.D.S., Deminicis B.B., Cezário A.S., Santos W.B.R.D. (2017). Aspects of Acidosis in Ruminants with a Focus on Nutrition: A Review. JAS.

[41] Hernández J., Benedito J.L., Abuelo A., Castillo C. (2014). Ruminal Acidosis in Feedlot: From Aetiology to Prevention. Sci. World J..

[42] Rabaza A., Banchero G., Cajarville C., Zunino P., Britos A., Repetto J.L., Fraga M. (2020). Effects of Feed Withdrawal Duration on Animal Behaviour, Rumen Microbiota and Blood Chemistry in Feedlot Cattle: Implications for Rumen Acidosis. Animal.

[43] Viana P.R.L., Viana L.F., Araújo G.H.M., de Moraes I.D.T., Queiroz P.J.B., Cagnini D.Q., da Silva L.A.F., Rabelo R.E. (2022). The Macroscopic and Microscopic Description of Ruminal Lesions in Feedlot Bovine. Ciênc. Anim. Bras..

[44] Bus J.D., Stockhofe N., Webb L.E. (2019). Abomasal Damage in Veal Calves. J. Dairy Sci..

[45] Braun U., Gerspach C., Hilbe M., Devaux D.J., Reif C. (2019). Clinical and Laboratory Findings in 60 Cows with Type-3 Abomasal Ulcer. Schweiz. Arch. Tierheilkd..

[46] Freitas S.L.R., Queiroz P.J.B., Fernandes J.J.R., Nascente E.P., Santos A.S., Nascimento K.S., Silva L.A.F. (2023). Occurrence of Clinical Laminitis after Adaptation to Confinement: Effects on Morphology, Density, and Mineral Composition of the Hoof of Nellore Cattle after Finishing. Pesq. Vet. Bras..

[47] Stefańska B., Komisarek J., Nowak W. (2020). Non-Invasive Indicators Associated with Subacute Ruminal Acidosis in Dairy Cows. Ann. Anim. Sci..

[48] Khorrami B., Khiaosa-ard R., Zebeli Q. (2021). Models to Predict the Risk of Subacute Ruminal Acidosis in Dairy Cows Based on Dietary and Cow Factors: A Meta-Analysis. J. Dairy Sci..

[49] Garrett E.F., Pereira M.N., Nordlund K.V., Armentano L.E., Goodger W.J., Oetzel G.R. (1999). Diagnostic Methods for the Detection of Subacute Ruminal Acidosis in Dairy Cows. J. Dairy Sci..

[50] Enemark J.M.D. (2008). The Monitoring, Prevention and Treatment of Sub-Acute Ruminal Acidosis (SARA): A Review. Vet. J..

[51] Li W., Gelsinger S., Edwards A., Riehle C., Koch D. (2019). Transcriptome Analysis of Rumen Epithelium and Meta-Transcriptome Analysis of Rumen Epimural Microbial Community in Young Calves with Feed Induced Acidosis. Sci. Rep..

[52] Krause K.M., Oetzel G.R. (2006). Understanding and Preventing Subacute Ruminal Acidosis in Dairy Herds: A Review. Anim. Feed Sci. Technol..

[53] Esposito G., Irons P.C., Webb E.C., Chapwanya A. (2014). Interactions between Negative Energy Balance, Metabolic Diseases, Uterine Health and Immune Response in Transition Dairy Cows. Anim. Reprod. Sci..

[54] Drong C., Meyer U., von Soosten D., Frahm J., Rehage J., Breves G., Dänicke S. (2016). Effect of Monensin and Essential Oils on Performance and Energy Metabolism of Transition Dairy Cows. J. Anim. Physiol. Anim. Nutr..

[55] Klein R., Nagy O., Tóthová C., Chovanová F. (2020). Clinical and Diagnostic Significance of Lactate Dehydrogenase and Its Isoenzymes in Animals. Vet. Med. Int..

[56] Delaby L., Faverdin P., Michel G., Disenhaus C., Peyraud J.L. (2009). Effect of Different Feeding Strategies on Lactation Performance of Holstein and Normande Dairy Cows. Animal.

[57] Mann S., Yepes F.A.L., Overton T.R., Wakshlag J.J., Lock A.L., Ryan C.M., Nydam D.V. (2015). Dry Period Plane of Energy: Effects on Feed Intake, Energy Balance, Milk Production, and Composition in Transition Dairy Cows. J. Dairy Sci..

[58] Stone W.C. (2004). Nutritional Approaches to Minimize Subacute Ruminal Acidosis and Laminitis in Dairy Cattle. J. Dairy Sci..

[59] Zebeli Q., Dijkstra J., Tafaj M., Steingass H., Ametaj B.N., Drochner W. (2008). Modeling the Adequacy of Dietary Fiber in Dairy Cows Based on the Responses of Ruminal pH and Milk Fat Production to Composition of the Diet. J. Dairy Sci..

[60] Lei M.A.C., Simões J. (2021). Invited Review: Ketosis Diagnosis and Monitoring in High-Producing Dairy Cows. Dairy.

[61] Mann S., McArt J., Abuelo A. (2019). Production-Related Metabolic Disorders of Cattle: Ketosis, Milk Fever and Grass Staggers. Practice.

[62] Fox F.H. (1971). Clinical Diagnosis and Treatment of Ketosis. J. Dairy Sci..

[63] Wu G., Bazer F.W., Lamb G.C., Wu G. (2020). Management of Metabolic Disorders (Including Metabolic Diseases) in Ruminant and Nonruminant Animals. Animal Agriculture.

[64] García A.M.B., Cardoso F.C., Campos R., Thedy D.X., González F.H.D. (2011). Metabolic Evaluation of Dairy Cows Submitted to Three Different Strategies to Decrease the Effects of Negative Energy Balance in Early Postpartum. Pesq. Vet. Bras..

[65] Roche J.R., Bell A.W., Overton T.R., Loor J.J. (2013). Nutritional Management of the Transition Cow in the 21st Century—A Paradigm Shift in Thinking. Anim. Prod. Sci..

[66] Mekuriaw Y. (2023). Negative Energy Balance and Its Implication on Productive and Reproductive Performance of Early Lactating Dairy Cows: Review Paper. J. Appl. Anim. Res..

[67] Cotter D.G., Ercal B., Huang X., Leid J.M., d’Avignon D.A., Graham M.J., Dietzen D.J., Brunt E.M., Patti G.J., Crawford P.A. (2014). Ketogenesis Prevents Diet-Induced Fatty Liver Injury and Hyperglycemia. J. Clin. Investig..

[68] Bradford B.J., Swartz T.H. (2020). Review: Following the Smoke Signals: Inflammatory Signaling in Metabolic Homeostasis and Homeorhesis in Dairy Cattle. Animal.

[69] Fukao T., Mitchell G., Sass J.O., Hori T., Orii K., Aoyama Y. (2014). Ketone Body Metabolism and Its Defects. J. Inherit. Metab. Dis..

[70] Luo Z., Yu S., Zeng W., Zhou J. (2021). Comparative Analysis of the Chemical and Biochemical Synthesis of Keto Acids. Biotechnol. Adv..

[71] Tufarelli V., Colonna M.A., Losacco C., Puvača N. (2023). Biological Health Markers Associated with Oxidative Stress in Dairy Cows during Lactation Period. Metabolites.

[72] Service F.J. (1995). Hypoglycemic Disorders. N. Engl. J. Med..

[73] Holtenius P., Holtenius K. (1996). New Aspects of Ketone Bodies in Energy Metabolism of Dairy Cows: A Review. J. Vet. Med. Ser. A.

[74] Roche J.R., Kay J.K., Friggens N.C., Loor J.J., Berry D.P. (2013). Assessing and Managing Body Condition Score for the Prevention of Metabolic Disease in Dairy Cows. Vet. Clin. Food Anim. Pract..

[75] Guliński P. (2021). Ketone Bodies—Causes and Effects of Their Increased Presence in Cows’ Body Fluids: A Review. Vet. World.

[76] Holzhauer M., Valarcher J.-F. (2024). Literature Review and Metanalysis of Fatty Liver Syndrome in Dairy Cows and Evaluation of Reference Values of Triacyl Glycerides in Liver and NEFA, BHB, Glucose and Insulin in Serum. Curr. Trends Intern. Med..

[77] Mauvais-Jarvis F., Sobngwi E., Porcher R., Riveline J.-P., Kevorkian J.-P., Vaisse C., Charpentier G., Guillausseau P.-J., Vexiau P., Gautier J.-F. (2004). Ketosis-Prone Type 2 Diabetes in Patients of Sub-Saharan African Origin: Clinical Pathophysiology and Natural History of β-Cell Dysfunction and Insulin Resistance. Diabetes.

[78] Mooli R.G.R., Ramakrishnan S.K. (2022). Emerging Role of Hepatic Ketogenesis in Fatty Liver Disease. Front. Physiol..

[79] Zhang G., Ametaj B.N. (2020). Ketosis an Old Story Under a New Approach. Dairy.

[80] Zhang Q., Guo X., Zheng M., Chen D., Chen X. (2021). Altering Microbial Communities: A Possible Way of Lactic Acid Bacteria Inoculants Changing Smell of Silage. Anim. Feed Sci. Technol..

[81] Vicente F., Rodríguez M.L., Martínez-Fernández A., Soldado A., Argamentería A., Peláez M., de la Roza-Delgado B. (2014). Subclinical Ketosis on Dairy Cows in Transition Period in Farms with Contrasting Butyric Acid Contents in Silages. Sci. World J..

[82] Duffield T. (2000). Subclinical Ketosis in Lactating Dairy Cattle. Vet. Clin. N. Am. Food Anim. Pract..

[83] Li K., Wang W., Wu J., Xiao W. (2023). β-Hydroxybutyrate: A Crucial Therapeutic Target for Diverse Liver Diseases. Biomed. Pharmacother..

[84] Auerbach H., Nadeau E. (2020). Effects of Additive Type on Fermentation and Aerobic Stability and Its Interaction with Air Exposure on Silage Nutritive Value. Agronomy.

[85] Schmitz R., Schnabel K., Frahm J., von Soosten D., Meyer U., Hüther L., Spiekers H., Rehage J., Sauerwein H., Dänicke S. (2021). Effects of Energy Supply from Roughage and Concentrates and the Occurrence of Subclinical Ketosis on Blood Chemistry and Liver Health in Lactating Dairy Cows during Early Lactation. Dairy.

[86] Issi M., Gül Y., Başbuğ O. (2016). Evaluation of Renal and Hepatic Functions in Cattle with Subclinical and Clinical Ketosis. Turk. J. Vet. Anim. Sci..

[87] Satoła A., Bauer E.A. (2021). Predicting Subclinical Ketosis in Dairy Cows Using Machine Learning Techniques. Animals.

[88] Wagner B., Gerletti P., Fürst P., Keuth O., Bernsmann T., Martin A., Schäfer B., Numata J., Lorenzen M.C., Pieper R. (2022). Transfer of Cannabinoids into the Milk of Dairy Cows Fed with Industrial Hemp Could Lead to Δ9-THC Exposure That Exceeds Acute Reference Dose. Nat. Food.

[89] Cascone G., Licitra F., Stamilla A., Amore S., Dipasquale M., Salonia R., Antoci F., Zecconi A. (2022). Subclinical Ketosis in Dairy Herds: Impact of Early Diagnosis and Treatment. Front. Vet. Sci..

[90] Młynek K., Głowińska B. (2020). The Relationship of Body Condition and Chewing Time with Body Weight, the Level of Plasma Cocaine and Amphetamine Regulated Transcript, Leptin and Energy Metabolites in Cows until Reaching the Lactation Peak. Acta Vet. Brno.

[91] Djoković R., Šamanc H., Ilić Z., Kurćubić V. (2009). Blood Glucose, Insulin and Inorganic Phosphorus in Healthy and Ketotic Dairy Cows after Intravenous Infusion of Glucose Solution. Acta Vet. Brno.

[92] Krempaský M., Maskaľová I., Bujňák L., Vajda V. (2014). Ketone Bodies in Blood of Dairy Cows: Prevalence and Monitoring of Subclinical Ketosis. Acta Vet. Brno.

[93] Serrenho R.C., Williamson M., Berke O., LeBlanc S.J., DeVries T.J., McBride B.W., Duffield T.F. (2022). An Investigation of Blood, Milk, and Urine Test Patterns for the Diagnosis of Ketosis in Dairy Cows in Early Lactation. J. Dairy Sci..

[94] Faruk M., Park B., Ha S., Lee S., Mamuad L., Cho Y. (2020). Comparative Study on Different Field Tests of Ketosis Using Blood, Milk, and Urine in Dairy Cattle. Vet. Med..

[95] Bellato A., Tondo A., Dellepiane L., Dondo A., Mannelli A., Bergagna S. (2023). Estimates of Dairy Herd Health Indicators of Mastitis, Ketosis, Inter-Calving Interval, and Fresh Cow Replacement in the Piedmont Region, Italy. Prev. Vet. Med..

[96] Wang L., Cen S., Wang G., Lee Y., Zhao J., Zhang H., Chen W. (2020). Acetic Acid and Butyric Acid Released in Large Intestine Play Different Roles in the Alleviation of Constipation. J. Funct. Foods.

[97] Pechová A., Nečasová A. (2018). The Relationship Between Subclinical Ketosis and Ruminal Dysfunction in Dairy Cows. Ann. Anim. Sci..

[98] Dhatariya K.K., Glaser N.S., Codner E., Umpierrez G.E. (2020). Diabetic Ketoacidosis. Nat. Rev. Dis. Primers.

[99] Enjalbert F., Nicot M.C., Bayourthe C., Moncoulon R. (2001). Ketone Bodies in Milk and Blood of Dairy Cows: Relationship between Concentrations and Utilization for Detection of Subclinical Ketosis. J. Dairy Sci..

[100] Steen A., Grønstøl H., Torjesen P.A. (1997). Glucose and Insulin Responses to Glucagon Injection in Dairy Cows with Ketotis and Fatty Live. J. Vet. Med. Ser. A.

[101] Borchardt S., Staufenbiel R. (2012). Evaluation of the Use of Nonesterified Fatty Acids and β-Hydroxybutyrate Concentrations in Pooled Serum Samples for Herd-Based Detection of Subclinical Ketosis in Dairy Cows during the First Week after Parturition. J. Am. Vet. Med. Assoc..

[102] Kumar V., Gill K.D. (2018). Qualitative Analysis of Ketone Bodies in Urine. Basic Concepts in Clinical Biochemistry: A Practical Guide.

[103] McArt J.A.A., Nydam D.V., Oetzel G.R. (2012). A Field Trial on the Effect of Propylene Glycol on Displaced Abomasum, Removal from Herd, and Reproduction in Fresh Cows Diagnosed with Subclinical Ketosis. J. Dairy Sci..

[104] Cervenka M.C., Wood S., Bagary M., Balabanov A., Bercovici E., Brown M.-G., Devinsky O., Di Lorenzo C., Doherty C.P., Felton E. (2021). International Recommendations for the Management of Adults Treated With Ketogenic Diet Therapies. Neurol. Clin. Pract..

[105] Melendez P., Arévalos A., Duchens M., Pinedo P., Melendez P., Arévalos A., Duchens M., Pinedo P. (2019). Effect of an Intraruminal Monensin Bolus on Blood β-Hydroxybutyrate, Peripartum Diseases, Milk Yield and Solids in Holstein Cows. Rev. Mex. Cienc. Pecu..

[106] Heuer C., Schukken Y.H., Jonker L.J., Wilkinson J.I.D., Noordhuizen J.P.T.M. (2001). Effect of Monensin on Blood Ketone Bodies, Incidenceand Recurrence of Disease and Fertility in Dairy Cows. J. Dairy Sci..

[107] Kumari A., Kumar Jain V., Kumar Nehra A., Kumar M., Sharma M., Kumar A., Gupta S., Singh Y. (2022). Assessment of Haematological and Biochemical Alterations in Recumbent Buffaloes. Biol. Rhythm Res..

[108] Lomb J., von Keyserlingk M.A.G., Weary D.M. (2020). Behavioral Changes Associated with Fever in Transition Dairy Cows. J. Dairy Sci..

[109] Felsenfeld A.J., Levine B.S. (2006). Milk Alkali Syndrome and the Dynamics of Calcium Homeostasis. Clin. J. Am. Soc. Nephrol..

[110] Goff J.P. (2014). Calcium and Magnesium Disorders. Vet. Clin. Food Anim. Pract..

[111] DeGaris P.J., Lean I.J. (2008). Milk Fever in Dairy Cows: A Review of Pathophysiology and Control Principles. Vet. J..

[112] Pechet M.M., Bobadilla E., Carroll E.L., Hesse R.H. (1967). Regulation of Bone Resorption and Formation: Influences of Thyrocalcitonin, Parathyroid Hormone, Neutral Phosphate and Vitamin D3. Am. J. Med..

[113] Kroll M.H. (2000). Parathyroid Hormone Temporal Effects on Bone Formation and Resorption. Bull. Math. Biol..

[114] Stevenson M.A., Williamson N.B., Hardon D.W. (1999). The Effects of Calcium Supplementation of Dairy Cattle after Calving on Milk, Milk Fat and Protein Production, and Fertility. N. Z. Vet. J..

[115] Goff J.P. (2008). The Monitoring, Prevention, and Treatment of Milk Fever and Subclinical Hypocalcemia in Dairy Cows. Vet. J..

[116] Seifi H.A., Kia S. (2017). Subclinical Hypocalcemia in Dairy Cows: Pathophysiology, Consequences and Monitoring. IJVST.

[117] Rérat M., Schlegel P. (2014). Effect of Dietary Potassium and Anionic Salts on Acid–Base and Mineral Status in Periparturient Cows. J. Anim. Physiol. Anim. Nutr..

[118] Goff J.P. (2000). Pathophysiology of Calcium and Phosphorus Disorders. Vet. Clin. N. Am. Food Anim. Pract..

[119] Kostadinović L. (2023). Hydroponic Feed and Quality in Sustainable Dairy Animal Production. J. Agron. Technol. Eng. Manag..

[120] Kocabagli N. (2018). Prevention of Milk Fever: A Herd Health Approach to Dairy Cow Nutrition. Arch. Anim. Husb. Dairy Sci..

[121] Murray R.D., Horsfield J.E., McCormick W.D., Williams H.J., Ward D. (2008). Historical and Current Perspectives on the Treatment, Control and Pathogenesis of Milk Fever in Dairy Cattle. Vet. Rec..

[122] Sun M., Wu X., Yu Y., Wang L., Xie D., Zhang Z., Chen L., Lu A., Zhang G., Li F. (2020). Disorders of Calcium and Phosphorus Metabolism and the Proteomics/Metabolomics-Based Research. Front. Cell Dev. Biol..

[123] Reinhardt T.A., Lippolis J.D., McCluskey B.J., Goff J.P., Horst R.L. (2011). Prevalence of Subclinical Hypocalcemia in Dairy Herds. Vet. J..

[124] Ramberg C.F., Johnson E.K., Fargo R.D., Kronfeld D.S. (1984). Calcium Homeostasis in Cows, with Special Reference to Parturient Hypocalcemia. Am. J. Physiol. Regul. Integr. Comp. Physiol..

[125] Venjakob P.L., Borchardt S., Heuwieser W. (2017). Hypocalcemia—Cow-Level Prevalence and Preventive Strategies in German Dairy Herds. J. Dairy Sci..

[126] Arechiga-Flores C.F., Cortés-Vidauri Z., Hernández-Briano P., Lozano-Domínguez R.R., López-Carlos M.A., Macías-Cruz U., Avendaño-Reyes L., Arechiga-Flores C.F., Cortés-Vidauri Z., Hernández-Briano P. (2022). Hypocalcemia in the Dairy Cow. Review. Rev. Mex. Cienc. Pecu..

[127] Oetzel G.R. (1988). Parturient Paresis and Hypocalcemia in Ruminant Livestock. Vet. Clin. N. Am. Food Anim. Pract..

[128] Caixeta L.S., Ospina P.A., Capel M.B., Nydam D.V. (2017). Association between Subclinical Hypocalcemia in the First 3 Days of Lactation and Reproductive Performance of Dairy Cows. Theriogenology.

[129] Wilhelm A.L., Maquivar M.G., Bas S., Brick T.A., Weiss W.P., Bothe H., Velez J.S., Schuenemann G.M. (2017). Effect of Serum Calcium Status at Calving on Survival, Health, and Performance of Postpartum Holstein Cows and Calves under Certified Organic Management. J. Dairy Sci..

[130] Fehlberg L.K., Pineda A., Cardoso F.C. (2022). Validation of 2 Urine pH Measuring Techniques in a Prepartum Negative Dietary Cation-Anion Difference Diet and the Relationship with Production Performance. JDS Commun..

[131] Amanlou H., Akbari A.P., Farsuni N.E., Silva-del-Río N. (2016). Effects of Subcutaneous Calcium Administration at Calving on Mineral Status, Health, and Production of Holstein Cows. J. Dairy Sci..

[132] Wilms J., Wang G., Doelman J., Jacobs M., Martín-Tereso J. (2019). Intravenous Calcium Infusion in a Calving Protocol Disrupts Calcium Homeostasis Compared with an Oral Calcium Supplement. J. Dairy Sci..

[133] Miltenburg C.L., Duffield T.F., Bienzle D., Scholtz E.L., LeBlanc S.J. (2016). Randomized Clinical Trial of a Calcium Supplement for Improvement of Health in Dairy Cows in Early Lactation. J. Dairy Sci..

[134] Oetzel G.R. (2017). Fresh Cow Metabolic Diseases: Old Myths and New Data. American Association of Bovine Practitioners Conference Proceedings.

[135] Wilkens M.R., Nelson C.D., Hernandez L.L., McArt J.A.A. (2020). Symposium Review: Transition Cow Calcium Homeostasis—Health Effects of Hypocalcemia and Strategies for Prevention. J. Dairy Sci..

[136] Martín-Tereso J., Verstegen M.W.A. (2011). A Novel Model to Explain Dietary Factors Affecting Hypocalcaemia in Dairy Cattle. Nutr. Res. Rev..

